# Zincophilic MOF Protective Layer for Stable Zinc Anodes in Zinc‐Ion Batteries

**DOI:** 10.1002/chem.202502217

**Published:** 2025-08-22

**Authors:** Shuang Liu, Mariam Maisuradze, Min Li, Qizhi Li, Neda Kazemi, Marco Giorgetti

**Affiliations:** ^1^ Department of Industrial Chemistry University of Bologna Via Piero Gobetti 85 40129 Bologna Italy; ^2^ Elettra Sincrotrone Trieste 34149 Trieste Italy

**Keywords:** aqueous zinc batteries, interface stability, zinc metal anodes, zincophilic layer

## Abstract

Aqueous zinc‐ion batteries (AZIBs) are under the spotlight due to their substantial potential, abundant natural resources, inherent safety, and high specific capacity. However, uncontrollable zinc dendrite growth and side reactions on the zinc surface hinder the application of ZIBs. In this article, a uniform copper‐based metal‐organic frameworks (MOFs) coating layer was fabricated on a zinc metal surface (CuZIF@Zn) to serve as a protective interface. Synchrotron X‐ray techniques were employed to study the electrode structure before and after cycling. With its special structure, the MOF interfacial layer acts as an effective barrier layer to isolate the zinc anode from water molecules and electrolyte, thereby suppressing interfacial side reactions and passivation. Additionally, the abundance of zincophilic sites in the CuZIF layer ensures uniform deposition of Zn^2+^ while effectively inhibiting the growth of dendritic structures. Consequently, the CuZIF@Zn//CuZIF@Zn symmetric batteries exhibited cycling stability over 1000 cycles at 1.0 mA cm^−2^, attributed to the protective layer. Furthermore, a full cell incorporating an MnO_2_ cathode maintained 189 mAh g^−1^ over 700 cycles, demonstrating remarkable long‐term cycling performance.

## 1 Introduction

Aqueous rechargeable batteries have attracted significant attention in sustainable energy storage.^[^ ^1^, ^2^, ^3^ ^]^ Among these, aqueous zinc‐ion batteries (AZIBs) are considered to be one of the most promising candidates. Zinc metal is widely used as an anode due to its suitable potential (‐0.76 V Vs. SHE), low cost (∼US$ 2.4 kg^−1^), and excellent stability in air and water atmospheres.^[^ ^4^, ^5^, ^6^ ^]^ Furthermore, AZIBs possess a high theoretical gravimetric (820 mAh g^−1^) and volumetric capacity (5855 mAh cm^−3^).^[^ ^7^ ^]^ Nevertheless, the metallic zinc anode still suffers from severe inherent problems, such as by‐products, Zn dendrites, and passivation, which significantly affect the coulombic efficiency (CE) and battery lifespan.^[^ ^8^, ^9^ ^]^ Even in mild electrolytes, the hydrogen evolution reaction (HER) increases the local pH, forming zinc basic sulfate by‐products from zinc ions in the alkaline environment, which leads to swelling and failure of batteries.^[^ ^10^ ^]^ Moreover, uncontrollable zinc dendrite growth often occurs due to non‐uniform charge density on the zinc anode surface, resulting in uneven nucleation and deposition processes.^[^ ^11^ ^]^ As cycling progresses, the Zn dendrites can further grow and pierce the brittle glass fiber separator, eventually causing short circuits. Furthermore, passivation on the zinc surface is caused by interfacial corrosion reactions involving water molecules, resulting in the formation of by‐products. Therefore, developing effective strategies to address these issues associated with zinc anodes is critically important.

Studies have shown that introducing zincophilic sites can promote initial nucleation and guide uniform zinc growth.^[^ ^12^, ^13^ ^]^ Various strategies have been proposed to address these challenges, primarily including zincophilic electrolyte optimization,^[^ ^13^, ^14^, ^15^ ^]^ zincophilic electrode construction,^[^ ^12^, ^13^, ^16^ ^]^ and the design of zincophilic interfacial layers.^[^ ^17^, ^18^ ^]^ On the electrolyte side, electrolyte additives have proven effective in inhibiting zinc dendrite growth.^[^ ^15^, ^19^ ^]^ For example, Liu et al. introduced a zincophilic organic polymer into the electrolyte, which effectively inhibited the Zn dendrite growth.^[^ ^20^ ^]^ However, their gradual consumption during cycling results in a deterioration of long‐term performance. Regarding zinc electrode construction, zinc can be deposited in layers by fabricating a deposition framework.^[^ ^2^, ^21^ ^]^ Recently, Zhao et al. developed a new zincophilic alloy matrix electrode using a one‐step electrochemical method, which promotes zinc nucleation.^[^ ^22^ ^]^ While this method often compromises battery energy density, it significantly increases the cost of ZIBs.^[^ ^13^, ^23^ ^]^ Notably, anodic coatings not only regulate the uniform distribution of Zn^2^⁺, but also prevent the direct contact between water molecules and the zinc surface. For instance, Sun et al. reported a uniform anti‐corrosive Cu/Zn metallic layer that inhibited chemical corrosion and demonstrated stable electrochemical performance for 648 hours.^[^ ^24^ ^]^ Wang's group designed zincophilic zinc hydroxyfluoride nanowires to modulate Zn^2+^ deposition and exhibited excellent CE of 98.8%.^[^ ^25^ ^]^ Fan et al. fabricated a zincophilic polyanionic hydrogel to ensure uniform ion flux, and the full cell demonstrated a high capacity of 176 mAh g^−1^ over 4000 cycles.^[^ ^26^ ^]^ Wang and colleagues developed a flexible zincophilic polypyrrole paper to maintain the flatness of the zinc surface and presented a long cycle life of over 1600 hours^[^ ^27^ ^]^. The above strategy demonstrates that these zincophilic layers exhibit a strong affinity for zinc ions, thereby facilitating the absorption of zinc ions. By changing the zinc interface, the interfacial electric field and ion distribution can be effectively adjusted, thus guiding the uniform deposition of zinc.

In particular, MOFs have been widely regarded as promising interfacial materials in the field of energy due to their unique porosity and large specific surface area. In recent years, in the field of AZIBs, they have been recognized as protective layers for regulating Zn^2+^ diffusion and deposition due to their abundance of zincophilic sites.^[^ ^28^ ^]^ Studies have demonstrated that their ordered porous structures and organic coordination sites contribute to the enhanced stability of zinc anodes.^[^ ^29^, ^30^, ^31^ ^]^ The zinc affinity layer aims to improve the adsorption and binding capacity of zinc to the zinc anode through surface modification. The uniform deposition of zinc is promoted by lowering the nucleation energy barrier and homogenizing the electric field distribution, thereby preventing the growth of zinc dendrites. For example, the microporous structure of hydrophilic UIO‐66 MOF creates a nanoscale wetting effect with zinc, forming a zincophilic interface, which reduces the charge transfer resistance.^[^ ^32^ ^]^ 2D MOF nanoflakes containing N and O zincophilic sites can uniformly disperse Zn^2+^, allowing zinc ions to be deposited onto the nanosheets from the bottom up.^[^ ^11^ ^]^

Among various MOF types, zeolite imidazolate frameworks have a leaf‐like morphology (ZIF‐L), which provides a higher specific surface area and exposes more active sites (e.g., N and O atoms).^[^ ^11^, ^33^ ^]^ Moreover, copper (Cu), as a zincophilic species, possesses strong adsorption affinity for Zn^2+^ ions.^[^ ^34^ ^]^ Herein, we developed an artificial protective layer on zinc foil (CuZIF@Zn) to guide nucleation and subsequent growth of Zn^2+^ deposits. Furthermore, in order to have a good comparison, CoZIF@Zn was synthesized in addition to Zn metal, as it exhibits a similar trend in regulating Zn plating behavior.^[^ ^35^ ^]^ As a result, a symmetrical cell with the CuZIF@Zn anode maintained stable cycling for over 1000 hours at 1 mA cm^−1^ and more than 800 hours at 5 mA cm^−2^. Moreover, the full cell employing a MnO_2_ cathode demonstrated great electrochemical performance.

## 2 Results and discussion

**Figure 1:**
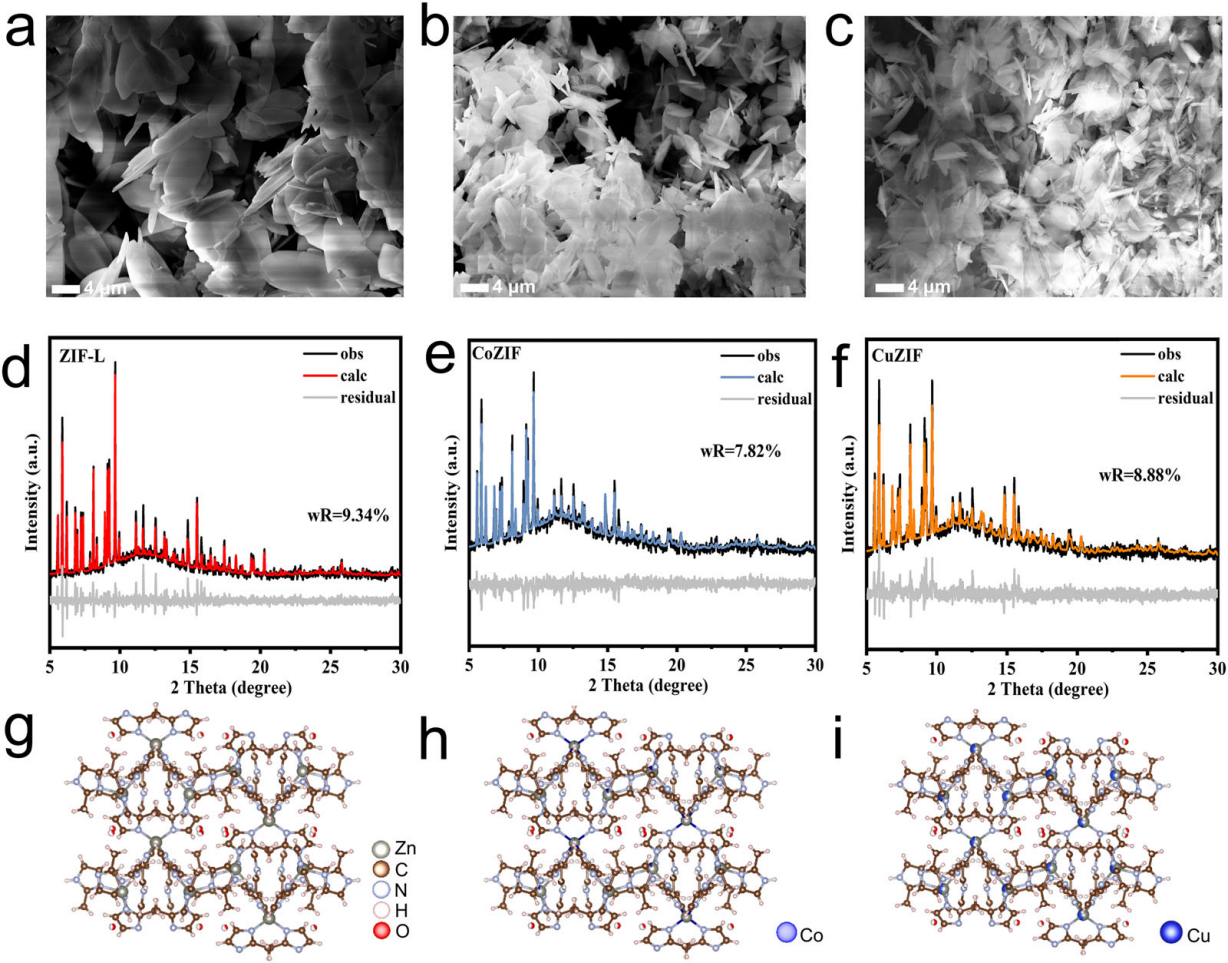
SEM images of a) ZIF‐L, b) CoZIF, and c) CuZIF. XRD patterns of d) ZIF‐L, e) CoZIF, and f) CuZIF. Crystal structure of g) ZIF‐L, h) CoZIF, and i) CuZIF phases.

The obtained ZIF‐L exhibits a typical leaf‐like morphology with a smooth surface structure, primarily composed of interconnected 2D nanosheets stacked together (Figure 1a). This unique structure not only provides a larger specific surface area but also facilitates electrolyte contact.^[^ ^36^ ^]^ The Zn:Cu and Zn:Co molar ratios are 3:1. Compared with ZIF‐L, the overall morphology of the CuZIF and CoZIF samples is largely retained (Figure 1b,c). Moreover, although the particle sizes of these samples decrease slightly, likely due to the subtle differences in their crystalline phases, the basic morphology is preserved. Powder X‐ray diffraction (PXRD) was performed on the synthesized materials to identify their crystal structure. Afterwards, Rietveld refinement was performed to obtain detailed structural parameters. The small residual variance factor (wR) indicates that the fitting results are reliable. As shown in Figure 1d, the pristine ZIF‐L powder crystallizes in an orthorhombic phase with a *Cmca* space group. Specifically, the unit cell parameters are *a *= 24.0663 Å, *b *= 16.9835 Å, and *c *= 19.6906 Å. Each Zn atom adopts a regular ZnN_4_ tetrahedral geometry, serving as the central metal ion. The crystallographic phase remains nearly unchanged after introducing Co and Cu into the framework (Figure 1e,f). A comparison of the XRD patterns of CuZIF@Zn, CoZIF@Zn, and pristine ZIF‐L reveals that Cu and Co incorporation do not disrupt the MOF structure, which is essential for maintaining ion pathways and structural integrity. As shown in the crystal structures in Figure 1g–i), most of the Cu ions are successfully incorporated into the ZIF‐L framework, while a smaller portion of Co ions occupy zinc sites. These results confirm that Cu effectively replaces Zn in the ZIF‐L structure and is more efficient than Co, indicating Cu's stronger ability to occupy Zn sites.

**Figure 2:**
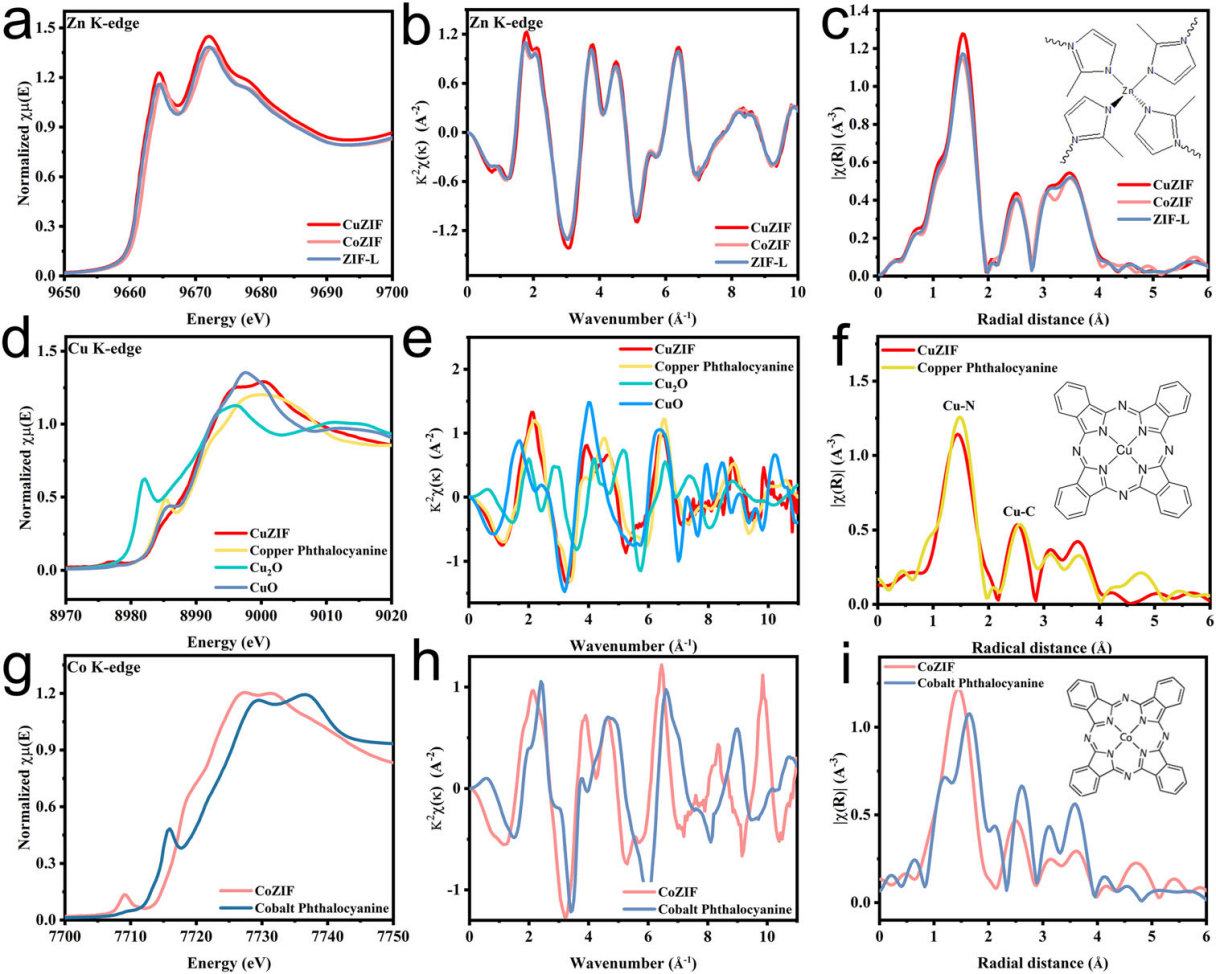
a) XANES spectra of Zn K‐edge of CuZIF, CoZIF, and ZIF‐L samples, b) *k* ^2^‐weighted EXAFS signals, c) corresponding Fourier transform (FT) signals. d) XANES spectra of Cu K‐edge of CuZIF, copper phthalocyanine, Cu_2_O, and CuO samples, e) *k* ^2^‐weighted EXAFS signals, f) corresponding FT signals. g) XANES spectra of Co K‐edge of CoZIF and cobalt phthalocyanine samples, h) *k* ^2^‐weighted EXAFS signals, i) corresponding FT signals.

X‐ray absorption spectroscopy (XAS) measurements were conducted to investigate the metal centers' chemical states and local coordination environments. The FT of the extracted *k* ^2^‐weighted extended X‐ray absorption fine structure (EXAFS) spectra at the Zn and Cu K‐edges reveals that CuZIF exhibits similar peak patterns and positions for both elements, suggesting that Zn and Cu share a common coordination environment (Figure S1a). This confirms that Cu successfully substitutes Zn within the ZIF framework, forming a 2‐methylimidazole copper complex (Figure S1b). Moreover, as shown in Figure 2a, neither the CuZIF nor the CoZIF samples exhibit significant changes in the Zn K‐edge XANES spectra, indicating that the local coordination geometry of Zn is preserved upon Cu and Co substitution. This observation is consistent with the XRD results. Further evidence is provided by the FT of the Zn K‐edge EXAFS spectra (Figure 2b,c), which shows only minor variations in peak intensity in the first coordination shell, likely due to structural disorder.^[^ ^37^ ^]^ These findings suggest that Zn maintains not only a similar oxidation state but also the local coordination environment in all three 2‐methylimidazole‐based frameworks.

Figure 2d presents the Cu K‐edge XANES spectrum of CuZIF in comparison with reference compounds. The absorption edge of CuZIF lies between those of CuO and Cu_2_O, suggesting that the average oxidation state of Cu in CuZIF falls between Cu^1+^ and Cu^2+^. The corresponding FT‐EXAFS spectrum (Figure 2e,f) shows peaks characteristic of copper phthalocyanine reference, Cu‐N peak at 1.47 Å and Cu‐C peak at 2.54 Å (uncorrected for phase‐shift). The presence of single Cu atoms coordinated to four N atoms indicates partial substitution of Zn by Cu within the ZIF framework. Furthermore, the FT‐EXAFS spectrum of CoZIF shows features similar to those of cobalt phthalocyanine, the first coordination shell of synthesized CoZIF appears to be more symmetric compared to that of cobalt phthalocyanine (Figure 2g–i). Overall, these results confirm that the Cu and Co atoms are dispersed within the 2‐methylimidazole and share similar coordination environments, forming a stable framework structure.

**Figure 3:**
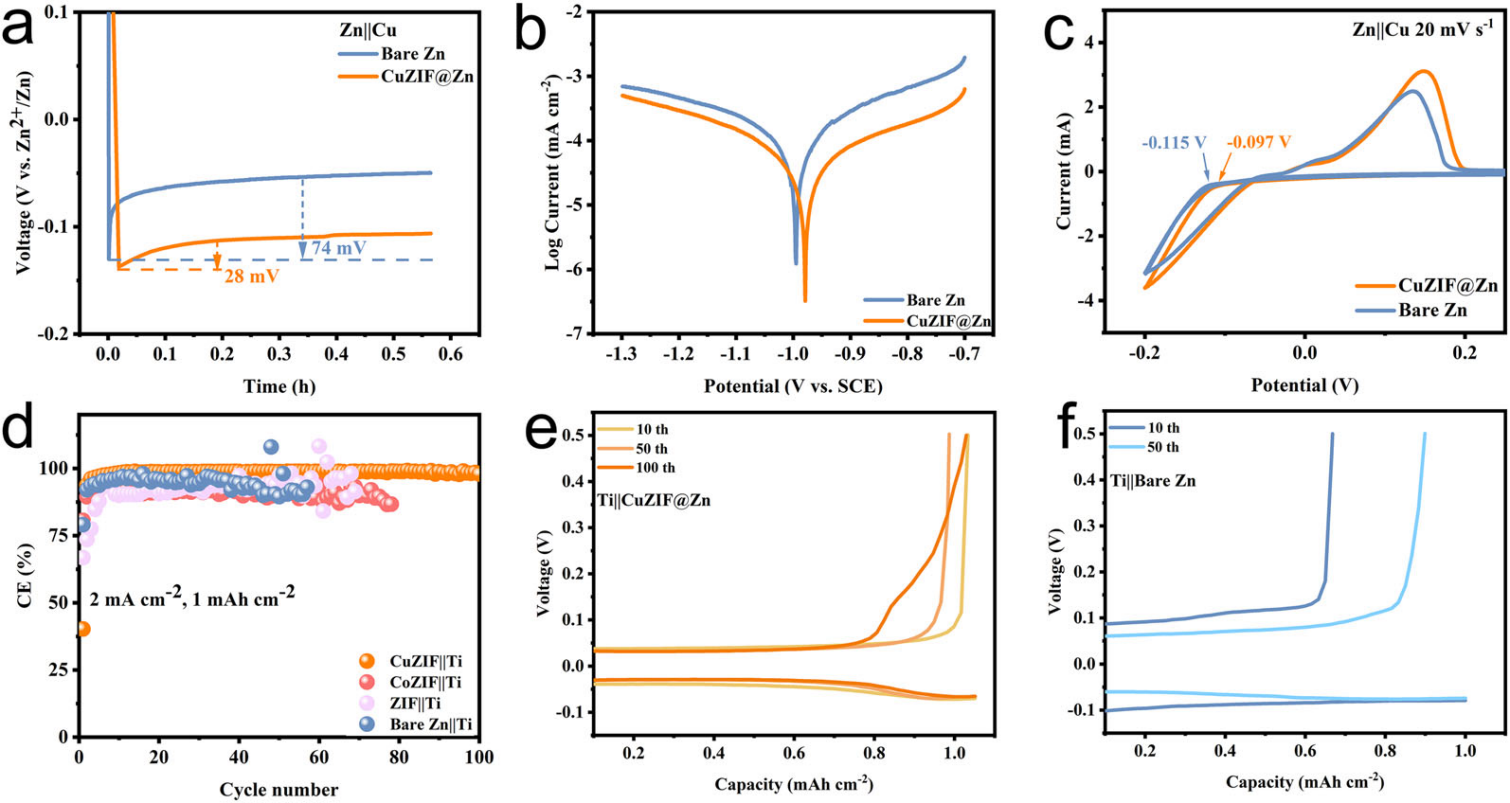
a) Nucleation overpotential curves of CuZIF@Zn and bare Zn under the current density of 1 mAh cm^−2^, b) Linear polarization curves of CuZIF@Zn and bare Zn, c) CV curves of CuZIF@Zn and bare Zn, d) CE of Zn plating/stripping at a current density of 2 mA cm^−2^ with an area capacity of 1 mAh cm^−2^. The voltage profiles of e) the CuZIF@Zn and f) bare Zn at various cycles.

Half cells were assembled using Cu foil electrodes to match the corresponding anodes, as Cu foil has good electrochemical stability and high electrical conductivity. As shown in Figure 3a, the nucleation overpotential was measured by applying a constant current density of 1.0 mA cm^−2^. The lower nuclear overpotential indicates a lower energy barrier for Zn deposition, resulting in more homogeneous Zn plating.^[^ ^13^ ^]^ Notably, the CuZIF@Zn anode exhibits a smaller nucleation overpotential compared to the bare Zn anode, confirming that CuZIF exhibits a strong affinity for Zn anode. By measuring the thickness of CuZIF@Zn coating, the nucleation overpotential at different thicknesses was compared (Figure S2). According to previous studies,^[^ ^35^, ^38^ ^]^ the difference in nucleation overpotential due to the thickness of the CuZIF@Zn layer is mainly due to the HER reaction in the mildly acidic ZnSO_4_ electrolyte, which leads to the local pH increase, the production of OH^−^, and the generation of Zn_4_(OH)_6_SO_4_·xH_2_O on the surface. This leads to the difference in electrochemical performance of the batteries. The introduction of zincophilic Cu sites by the CuZIF layer on the Zn anode strengthens the interfacial interaction between Zn^2^⁺ ions and the electrode, effectively reducing the energy barrier for zinc nucleation and growth.^[^ ^39^ ^]^ Furthermore, the corrosion resistance of the electrodes was evaluated using linear polarization measurements in a three‐electrode setup with 3 M ZnSO_4_ as the electrolyte, Zn or CuZIF@Zn as the working electrode, Cu foil as the counter electrode, and Saturated Calomel Electrode (SCE) as the reference electrode. As shown in Figure 3b, the linear polarization curves for CuZIF@Zn and bare Zn electrodes reveal that CuZIF@Zn exhibits a significantly higher corrosion potential (−0.976 vs. −0.993 V vs. SCE) and a lower corrosion current than bare Zn. These results indicate enhanced corrosion resistance and a reduced corrosion rate, suggesting that the CuZIF layer can act as an effective protective barrier by lowering the current density associated with water decomposition.

Cyclic voltammetry (CV) was conducted to evaluate the Zn plating/stripping behavior on the Cu substrate (Figure 3c). Compared to bare Zn (−0.115 V), CuZIF@Zn enables the reduction of Zn^2+^ ions to occur at a more positive potential of (−0.097 V), suggesting enhanced nucleation behavior. Moreover, CuZIF@Zn demonstrates a higher peak current density and a larger enclosed CV area compared to bare Zn, indicating improved electrochemical kinetics. Additionally, a more positive onset potential for Zn^2^⁺ reduction is observed for CuZIF@Zn, implying an increased density of nucleation sites.^[^ ^40^ ^]^ This facilitates uniform Zn deposition, and mitigates dendritic growth during cycling. In order to assess the reversible behavior of the Zn anodes in the electrochemical process, Ti foil was employed as the counter electrode to minimize side reactions from Cu foil in aqueous electrolytes. As shown in Figure 3d, Zn plating/stripping CE was measured at 2 mA cm^−2^, CuZIF@Zn delivers a stable average CE of ∼98% for 100 cycles. In contrast, CoZIF@Zn, ZIF@Zn, and bare Zn exhibit pronounced CE fluctuations after 50 cycles, indicating unstable Zn plating/stripping behavior on Ti foil. Furthermore, the CuZIF@Zn half‐cell maintains a consistently small voltage gap throughout cycling, suggesting excellent reversibility and a lower potential barrier in the phase transition between Zn^2+^ and Zn metal during cycling^[^ ^41^ ^]^ (Figure 3e,f). By comparison, bare Zn shows significantly higher voltage hysteresis than ZIF@Zn and CoZIF@Zn (Figure S3). These results collectively demonstrate that the CuZIF@Zn electrode enables faster plating kinetics, facilitating efficient and uniform Zn plating/stripping during prolonged cycling. Contact angle measurements further investigated the wettability of the surface. As shown in Figure S4, the initial contact angle of the CuZIF layer is larger than that of bare Zn, but it gradually becomes smaller with time. The contact angle indicates that CuZIF@Zn has a better wetting surface, which can enhance the conductivity of ions.

**Figure 4:**
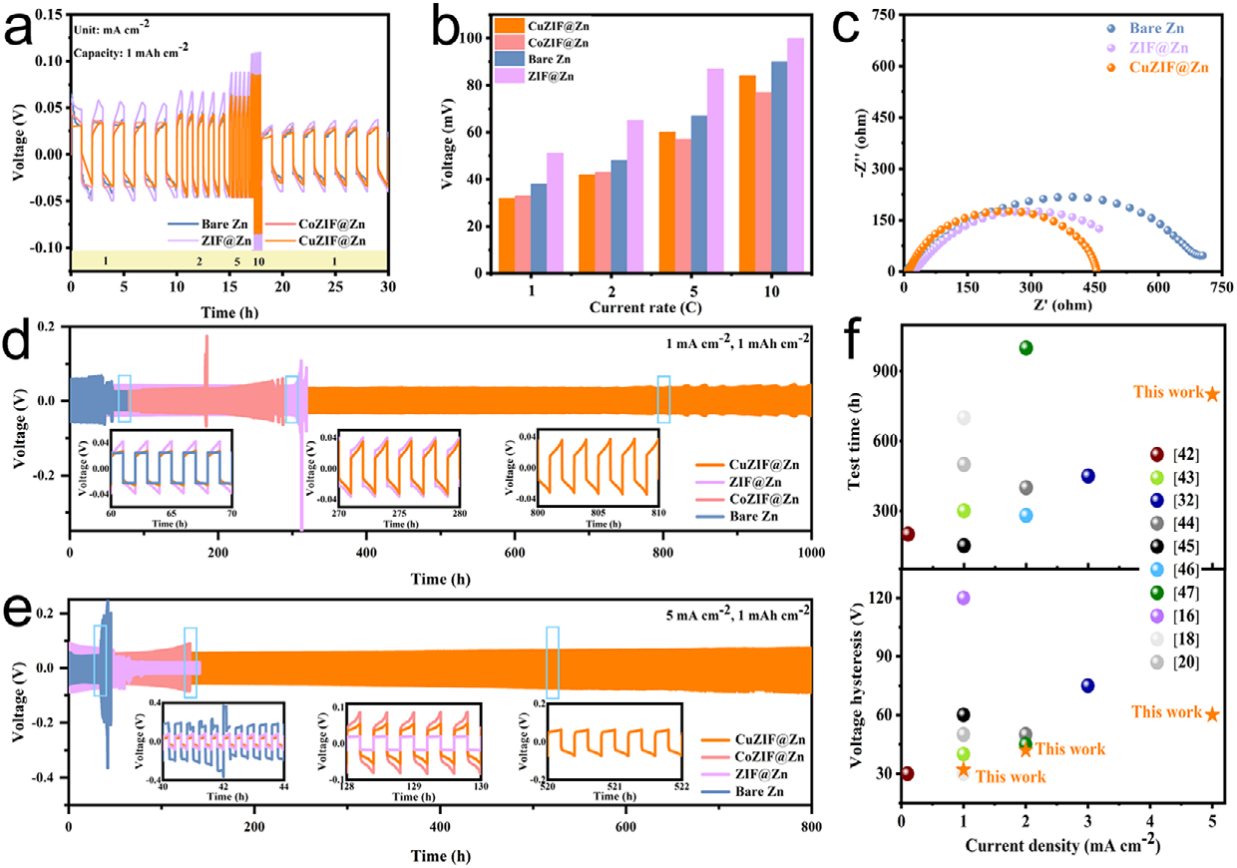
Symmetric cell performance of Zn//Zn cells using bare Zn, CuZIF@Zn, CoZIF@Zn, and ZIF@Zn, respectively. a) Rate performance at current densities of 1 mA cm^−2^ with an area capacity of 1 mAh cm^−2^, b) Polarization voltage between different rates, c) Nyquist plots of symmetric cells with different anodes, d) Cycling performance of symmetric cells with different anodes at current densities of 1 mA cm^−2^, e) Cycling performance of symmetric cells with different anodes at current densities of 5 mA cm^−2^, f) Comparisons of voltage hysteresis and cycling life in the literatures.

The anode performance of CuZIF@Zn was further evaluated using symmetric CuZIF@Zn// CuZIF@Zn cells in Figure 4a. As the current density increased from 1 to 10 mA cm^−2^, the CuZIF@Zn anode showed lower polarization compared to bare Zn. The voltage hysteresis values on Figure 4b indicate that the smaller polarization reflects the improved redox reaction kinetics enabled by CuZIF@Zn anode, as discussed previously. To analyze the charge transfer characteristics, electrochemical impedance spectroscopy (EIS) was conducted on the symmetric cells. As depicted in Figure 4c, CuZIF@Zn exhibits a lower charge transfer resistance (R_ct_) compared to the bare Zn, suggesting that the CuZIF coating possesses excellent Zn^2+^ affinity properties and acts as a hydrophilic electrolyte reservoir. Thus, it promotes the transport of Zn^2^⁺ ions and facilitates charge transfer.^[^ ^28^ ^]^

The bare Zn anode demonstrated the shortest cycle life of only 50 hours at 1.0 mA cm^−2^ (Figure 4d). Both ZIF@Zn and CoZIF@Zn extended the cycle life slightly, lasting up to 300 hours, after which a rapid voltage change occurred, indicating battery failure due to the short‐circuiting. The sudden drop in polarization voltage is attributed to the dynamic Zn dendrite growth that pierces the separator, causing internal short circuits. Concurrently, post‐cycling inspection revealed bulging on bare Zn anodes, due to the competitive HER (Figure S5).

Remarkably, the CuZIF@Zn anode exhibited a significantly prolonged cycle life, exceeding 1000 hours. The enhanced reversibility of CuZIF@Zn is ascribed to the CuZIF protective layer, which suppresses side reactions, lowers the nucleation barrier, and provides more sites for Zn^2^⁺ ions. At a higher current density of 5 mA cm^−^^2^, the CuZIF@Zn anode remained stable after 800 hours of cycling, whereas the CoZIF@Zn, ZIF@Zn, and bare Zn anodes short‐circuited and failed much earlier (Figure 4e). The accelerated failure at high current densities is mainly due to the enhanced dendrite growth and intensified side reactions. Compared to CoZIF@Zn, CuZIF@Zn shows a longer cycle life and lower voltage hysteresis at high current density, demonstrating superior interfacial stability. Figure 4f summarizes the cycle life and voltage hysteresis of these symmetric cells, highlighting that CuZIF coating provides outstanding advantages over other surface‐modified Zn anodes reported in the literature.^[^ ^16^, ^18^, ^20^, ^32^, ^42^, ^43^, ^44^, ^45^, ^46^, ^47^ ^]^

## 3 Results and discussion

**Figure 5:**
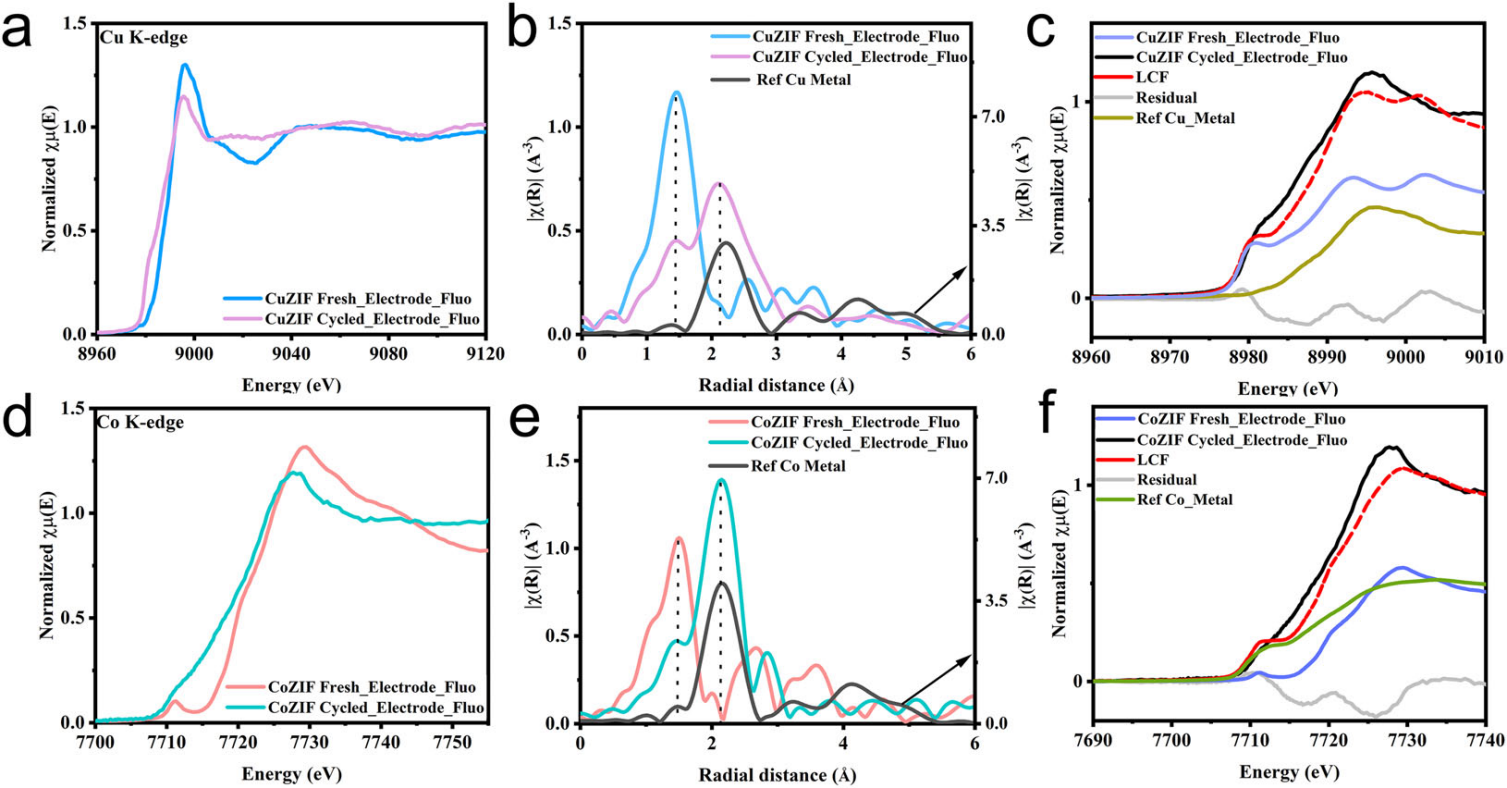
a) XANES spectra of fresh and cycled CuZIF electrode, b) Corresponding FTs of *k* ^2^‐weighted EXAFS, c) XANES linear combination fitting (LCF) analysis. d) XANES spectra of fresh and cycled CoZIF electrode, e) Corresponding FTs of *k* ^2^‐weighted XANES, f) XANES LCF analysis.

To gain insight into the structural evolution of the electrodes after cycling, XANES of Cu K‐edge and EXAFS for the cycled electrodes were collected in the fluorescence detection mode (Figure 5a,b). The first shell contribution of the fresh CuZIF@Zn electrode, similar to that of the powder samples, corresponds to the Cu‐N coordination. After cycling, a primary Cu‐N peak is observed at 1.47 Å with decreased intensity, along with the emergence of a new peak at 2.2 Å, which shares the same position as Cu metal and corresponds to the Cu‐Cu coordination, suggesting that Cu may have been partially reduced to the metallic phase. To further investigate the chemical state of Cu in the cycled electrode, a LCF was performed, as shown in Figure 5c. The cycled CuZIF electrode shows the ratio of CuZIF fresh electrode and Cu reference metal as 36% versus 64%, showing that 36% of Cu in the cycled electrode material remains in the original CuZIF form. A similar analysis was conducted for CoZIF (Figure 5d–f). The Co–N peak also shifted to a Co–Co peak after cycling. The fitting results indicate that 45% of CoZIF is retained in the Co–N coordination post‐cycling.

**Figure 6:**
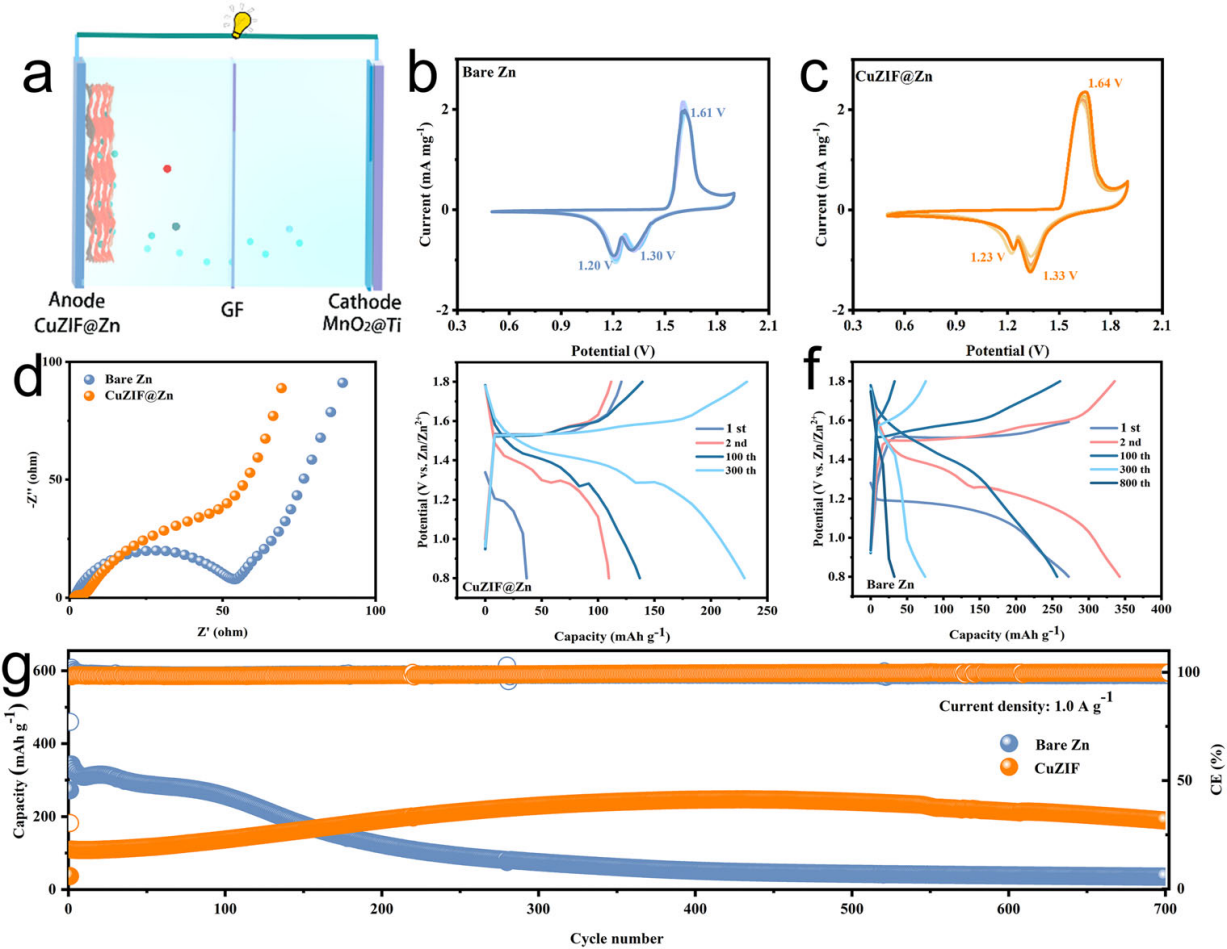
Full cell performance of Zn//MnO_2_ cells with different anodes. a) Schematic diagram of the full battery with CuZIF@Zn anode and MnO_2_ cathode, b) CV curve of bare Zn, c) CV curve of CuZIF@Zn, d) EIS plots of fresh cell, e) Charge‐discharge curves of CuZIF@Zn, f) Charge‐discharge curves of bare Zn, g) Long‐term cycling performance at current density of 1.0 A g^−1^.

A full battery was assembled to further evaluate the CuZIF@Zn anode, with MnO_2_ used as the cathode (Figure 6a). According to Figure S6, the XRD pattern of the synthesized MnO_2_ matches well with the standard diffraction data of α‐MnO_2_ (PDF #72–1982), confirming the successful synthesis of the α‐phase. CV curves recorded between 0.8 and 1.9 V (Figure 6b,c) exhibit distinct and well‐defined redox peaks, indicating a reversible conversion reaction between MnO_2_ and MnOOH, which is in agreement with literature reports.^[^ ^48^, ^49^, ^50^ ^]^ The highly overlapping CV profiles over multiple cycles indicate that the CuZIF coating does not induce any side reactions during the electrochemical processes. Notably, the CuZIF@Zn//MnO_2_ cell shows higher current responses compared to the bare Zn//MnO_2_ counterpart, suggesting the fast kinetics of Zn deposition/dissolution. Two reduction peaks of CuZIF@Zn at 1.33 V and 1.23 V are slightly higher than those of bare Zn at 1.30 V and 1.20 V. This is related to the rapid insertion process of Zn^2+^ and H^+^, which is attributed to the CuZIF protective layer. The EIS plots (Figure 6d) further confirm this, with the CuZIF@Zn//MnO_2_ cell exhibiting a smaller R_ct_ than the bare Zn//MnO_2_ cell, facilitating interfacial charge transfer and aligning well with the observations from the symmetric cell measurements.^[^ ^51^ ^]^

Additionally, long‐term cycling performance was evaluated at a current density of 1.0 A g^−1^. The corresponding charge‐discharge curves (Figure 6e,f) present two characteristic voltage plateaus, in accordance with the redox peaks observed in the CV curves. As shown in Figure 6g, due to the Zn^2+^ intercalation process is activated, the CuZIF@Zn capacity gradually increases during the initial cycle. More importantly, the CuZIF@Zn//MnO_2_ cell exhibits significantly enhanced cycling performance, maintaining a high specific capacity of approximately 189 mAh g^−1^ after 700 cycles. In contrast, the bare Zn//MnO_2_ cell exhibits rapid capacity decay, dropping to only ∼20 mAh g^−1^ over the same period. The superior electrochemical performance of the CuZIF@Zn//MnO_2_ system is attributed to the CuZIF protective layer, which inhibits dendrite growth and side reactions, ensuring long‐term stability and electrochemical reversibility.

## 4 Conclusion

In conclusion, we have developed a copper‐based MOF as a zinc anode protective layer via a simple solution method for AZIBs. Synchrotron XRD and XAS data show that substituting Cu and Co atoms for some Zn sites into ZIF‐L does not alter the framework structure. More importantly, zincophilic sites guide the uniform deposition of Zn^2+^ and suppress dendrite production due to their large nucleation sites. As a result, the CuZIF@Zn//CuZIF@Zn symmetric cell shows lower polarization voltage compared to the comparison sample over 1000 hours. Impressively, the symmetric cell can keep cycling for 800 hours even at a large current density of 5 mA cm^−2^ with an area capacity of 1.0 mAh cm^−2^. Moreover, the full cell with MnO_2_ cathode exhibits a stable cycling life with 189 mAh g^−1^ after 700 cycles at 1.0 A g^−1^. This study presents a new perspective on the strategy of adding zincophilic sites to MOFs as a protective layer for emerging zinc‐ion water batteries.

## Supporting information

The Supporting Information includes all experimental methods in detail and experimental data.

## Conflict of Interest

The authors declare no conflict of interest.

## Supporting information



Supporting Information
