# Supplementary material for: Zincophilic MOF Protective Layer for Stable Zinc Anodes in Zinc‐Ion Batteries
Source: Chemistry. 2025 Aug 22;31(57):e02217. doi: 10.1002/chem.202502217 (PMC12520058; doi:10.1002/chem.202502217)
Supplement: Supplementary file 1 — Supporting Information [file CHEM-31-e02217-s001.docx]

**Zincophilic MOF Protective Layer for Stable Zinc Anodes in Zinc-Ion Batteries**

**Shuang Liu^1^, Mariam Maisuradze^1^, Min Li^2^, Qizhi Li^1^, Neda Kazemi^1^, and Marco Giorgetti^1 *^**

1 Department of Industrial Chemistry, University of Bologna, 40129 Bologna, Italy

2 Elettra Sincrotrone Trieste, 34149 Trieste, Italy

*Corresponding author

E-mail addresses: [marco.giorgetti@unibo.it](mailto:marco.giorgetti@unibo.it)

**Experimental Section**

All chemicals were used without further purification and purchased from Sigma-Aldrich.

1. Synthesis of ZIF-L sample

Specifically, the Zn(NO_3_)_2_·6H_2_O (0.6 g) was dissolved in distilled water (40 mL). Then 2-methylimidazole (1 g) was dispersed into distilled water (40 mL). The two solutions were mixed, followed by stirring until mixed well. Subsequently, the mixture was placed to an oven and kept at 35 ℃ for 4 h to allow aging.

1. Synthesis of CoZIF sample

This process follows the same method as the ZIF-L synthesis. Firstly, the Zn(NO_3_)_2_·6H_2_O (0.6 g), Co(NO_3_)_2_·6H_2_O (0.1986 g), and distilled water (40 mL) were added into a beaker. Then 2-methylimidazole (1 g) was dispersed into distilled water (40 mL). The two solutions were mixed, followed by stirring until mixed well. Subsequently, the mixture was placed to an oven and kept at 35 ℃ for 4 h to allow aging.

1. Synthesis of CuZIF sample

This process follows the same method as the CoZIF synthesis. Firstly, the Zn (NO_3_)_2_·6H_2_O (0.6 g), Cu(NO_3_)_2_·6H_2_O (0.1958 g), and distilled water (40 mL) were added into a beaker. Then 2-methylimidazole (1 g, Sigma-Aldrich) was dispersed into distilled water (40 mL). The two solutions were mixed, followed by stirring until mixed well. Subsequently, the mixture was placed to an oven and kept at 35 ℃ for 4 h to allow aging.

1. Synthesis of α-MnO_2_ sample

Specifically, 2 mL of 0.5 M H_2_SO_4_ (98%), 60 mL of 0.05 M MnSO_4_·H_2_O (99%) were added into a beaker under vigorous stirring. Then, 20 mL of 0.1 M KMnO_4_ (99.5%) was dissolved into the solution slowly. Afterward, the mixture was put into an oven at 120 ℃ for 12 h. Ultimately, the brown precipitate was separated via centrifugation, thoroughly washed with distilled water, and then dried for 12 h.

**Electrochemical measurements**

All the coin cell test was conducted in 2032-coin cells. The working electrode was prepared by mixing the active material (70%), carbon black (20%), and PVDF (10%) and grinding for 30 min mixed with NMP. Then the slurry material was spread on the Zn sheet. Zn//Cu and Zn//Ti half cells design: The half cell was assembled from the bare Zn foil or CuZIF@Zn electrodes, 3 M ZnSO_4_ electrolyte, glass fiber separator, and Cu foil or Ti foil. All cells were assembled in open-air conditions and aged for 10 h before performing electrochemical measurements. For Zn//Zn symmetric cells used Zn foil as electrodes, glass-fiber membrane as the separator and 3 M ZnSO_4_ as the electrolyte. For full cells used MnO_2_@Ti as the cathode, a glass-fiber membrane as the separator and 2 M ZnSO_4_/0.2 M MnSO_4_ as the electrolyte. Cyclic voltammetry (CV) was performed by means of CH instruments model 660. Linear polarization curves measurements were carried out in a three-electrode setup with 3 M ZnSO_4_ as the electrolyte, Zn or CuZIF@Zn as the working electrode, Cu foil as the counter electrode, and Saturated Calomel Electrode (SCE) as the reference electrode at the CHI660E electrochemical workstation. The galvanostatic charge/discharge tests were performed using NEWWARE environmental test chamber MHW-25.

**Materials** **characterization**

The morphology of the samples was investigated by scanning electron microscope (SEM, ZEISS crossbeam 350). XRD data were recorded by using a monochromatic X-ray beam (wavelength of 0.827 Å) at the MCX beamline in Elettra synchrotron Trieste (Italy). The storage ring operated at 2.0 GeV in top up mode with a typical current of 300 mA. PXRD data of the powder samples were collected in a capillary geometry, setting the spinner at 3000. The crystal structure was refined using GSAS-II^1^. The X-ray absorption fine structure spectra were conducted at beamline XAFS^2^ of Elettra Synchrotron Trieste. Data were recorded at the Zn (9660 eV), Co (7709 eV) and Cu K-edge (8979 eV) in transmission mode for powder samples, and using ionization chambers filled with a mixture of Ar, N_2_, and He in order to have 10%, 70%, and 95% of absorption in the I0, I1, and I2 chambers. An internal reference of zinc, cobalt and copper foil was used for energy calibration in each scan. This allowed a continuous monitoring of the energy during consecutive scans. No energy drifts of the monochromator were observed during the experiments. Fluorescence modality was used for the electrodes. The white beam was monochromatized using a fixed exit monochromator equipped with a pair of Si(111) crystals. XAS data treatment was conducted using the Athena program^3^. XANES spectra were normalized to an edge jump of unity. A prior removal of the background absorption was done by subtraction of a linear function extrapolated from the pre-edge region.

1. Toby, B. H.; Von Dreele, R. B., GSAS-II: the genesis of a modern open-source all purpose crystallography software package. Journal of Applied Crystallography 2013, 46 (2), 544-549.

2. Aquilanti, G.; Giorgetti, M.; Dominko, R.; Stievano, L.; Arčon, I.; Novello, N.; Olivi, L., Operandocharacterization of batteries using x-ray absorption spectroscopy: advances at the beamline XAFS at synchrotron Elettra. Journal of Physics D: Applied Physics 2017, 50 (7).

3. Ravel, B.; Newville, M., ATHENA,ARTEMIS,HEPHAESTUS: data analysis for X-ray absorption spectroscopy usingIFEFFIT. Journal of Synchrotron Radiation 2005, 12 (4), 537-541.


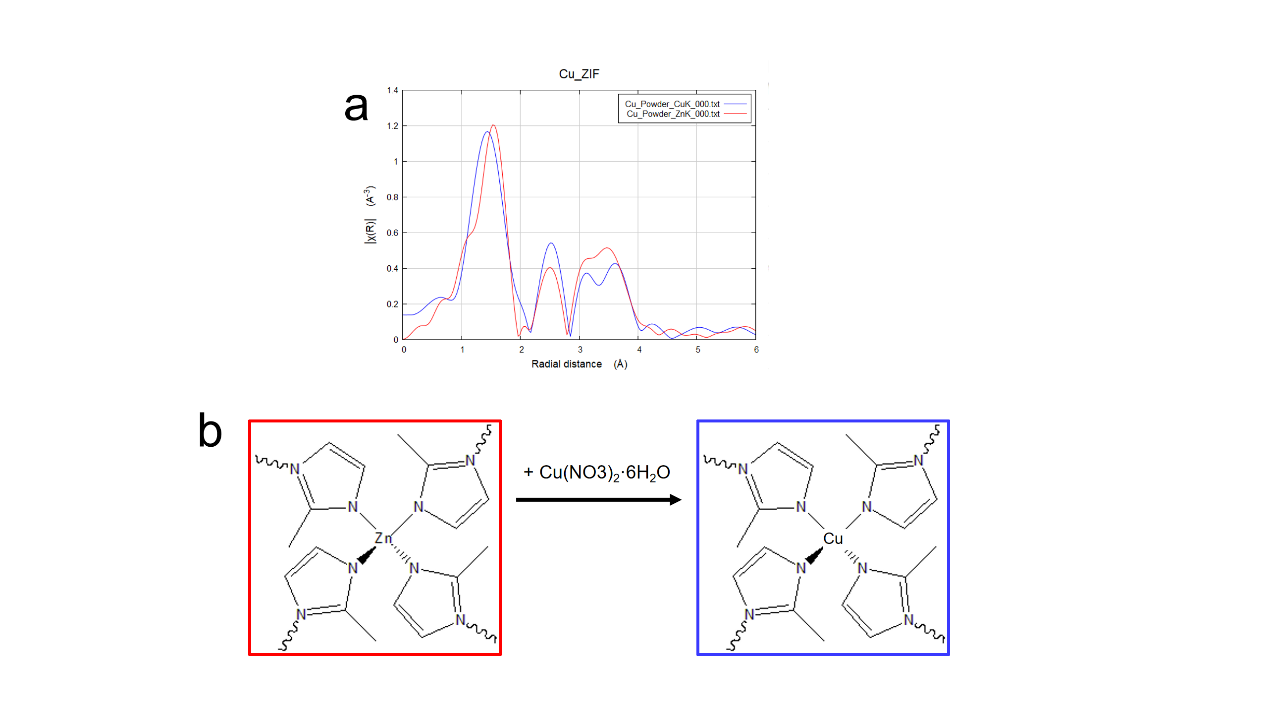


Fig. S1. (a) Fourier transform signals of CuZIF powder. (b) Schematic diagram of the substitution of Cu atoms.


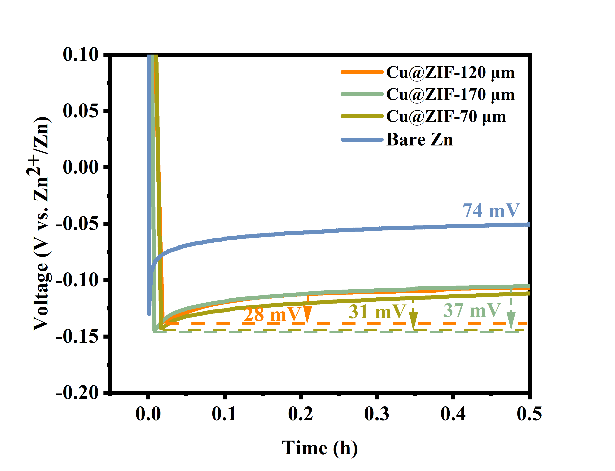


Fig. S2. Nucleation overpotential of different electrodes under the current density of 1 mA cm^-2^.


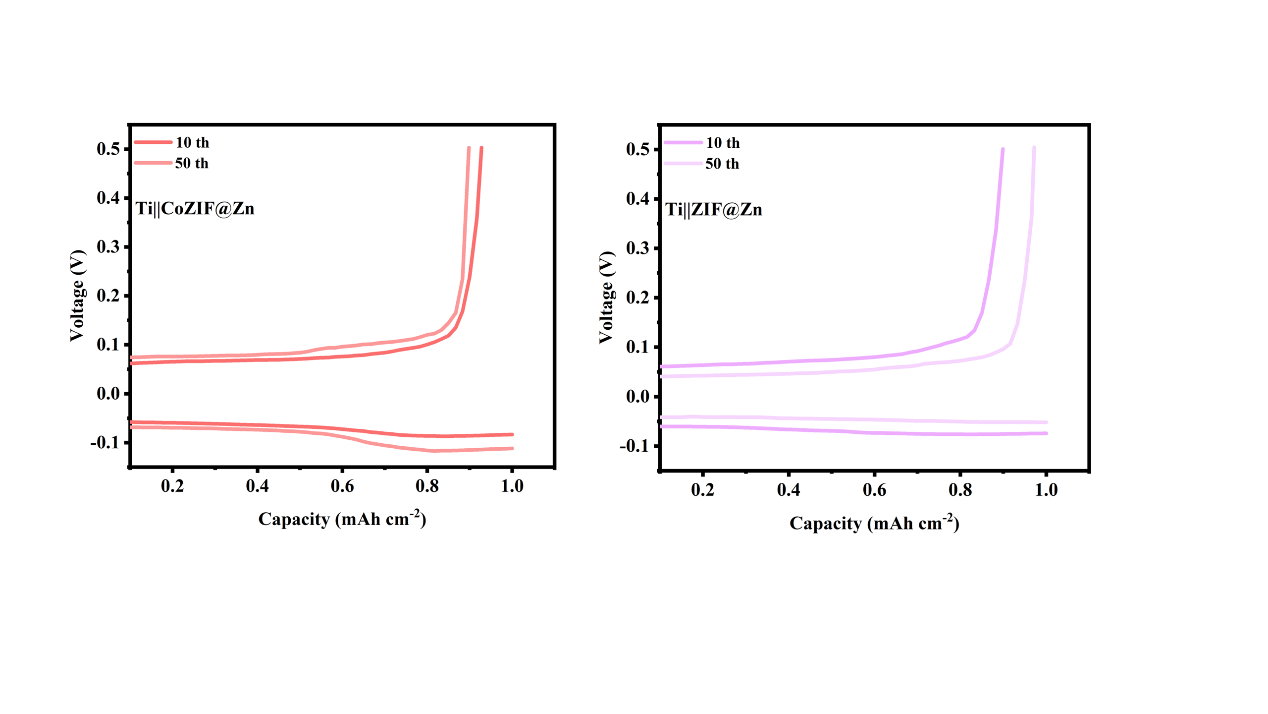


Fig. S3. The voltage profiles of Ti||CoZIF@Zn and Ti||ZIF@Zn cell.


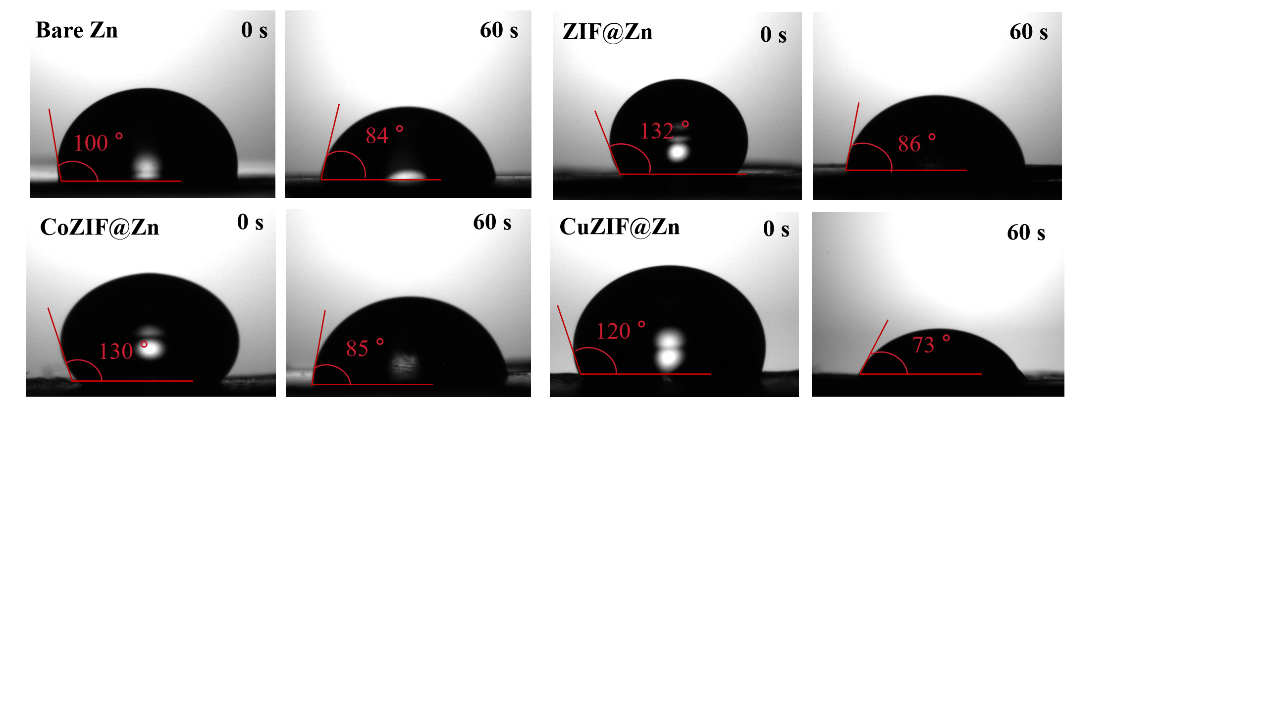


Fig. S4 Contact angle of 3 M ZnSO_4_ droplet on different electrodes.


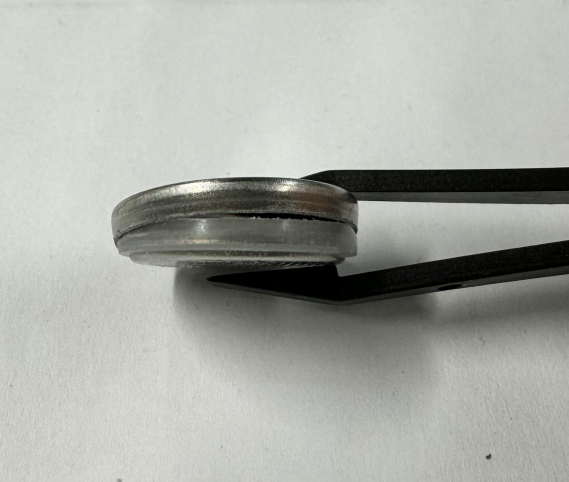


Fig. S5. After cycled of Zn//Zn symmetric cell.


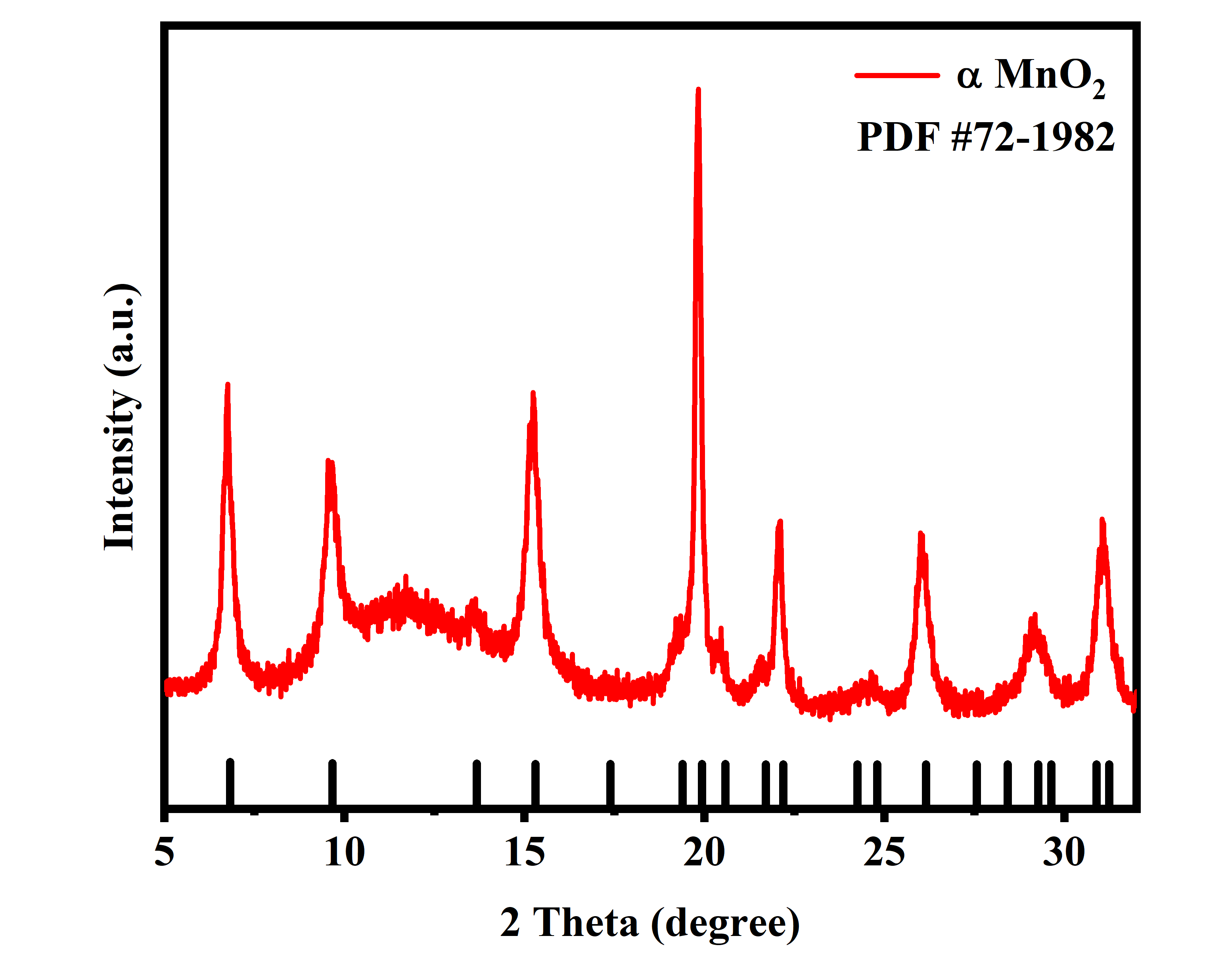


Fig. S6. XRD pattern of α-MnO_2_.
